# Supplementary material for: Are carbon emissions trading and green financial instruments synergistic? -Comprehensive quantitative research based on content analysis
Source: PLoS One. 2024 Mar 7;19(3):e0298601. doi: 10.1371/journal.pone.0298601 (PMC10919601; doi:10.1371/journal.pone.0298601)
Supplement: S1 Data — (ZIP) [file pone.0298601.s001.zip › Data/地级市Z_F工作报告.zip!/╕ú╜¿/┴·╤╥╩╨/└┤╫╘╣1⁄2╓┌║┼í╛┬φ┐╦╩2╛▌═°í┐_2015.docx]

各位代表：

　　现在，我代表龙岩市人民政府向大会作政府工作报告，请予审议，并请市政协委员和其他列席的同志提出意见。

　　一、2014年工作回顾

　　过去一年，在省委、省政府和市委的正确领导下，全市各级政府认真贯彻落实党的十八大、十八届三中、四中全会精神，深入学习贯彻习近平总书记系列重要讲话和来闽来岩考察重要讲话精神，牢牢把握中央支持福建加快发展、支持原中央苏区振兴发展的重大机遇，全力以赴稳增长、促改革、调结构、惠民生，全市经济社会发展取得新成效。预计实现地区生产总值1618亿元，增长9.7%；公共财政总收入261.7亿元，增长5%，其中地方公共财政收入119.8亿元，增长2.2%；全社会固定资产投资1590亿元，增长22.4%；外贸出口总值24.2亿美元，增长14%；实际利用外资2.4亿美元、增长11.5%，内联引资实际利用市外资金178亿元、增长13%；社会消费品零售总额548亿元，增长14.1%；居民消费价格总水平上涨1.9%；城镇居民人均可支配收入26153元，增长9.9%；农民人均可支配收入12055元，增长11.2%；城镇登记失业率2.5%；人口自然增长率9.8‰；年度节能减排目标预计可以完成。

　　一年来的主要工作和成效是：

　　（一）全力应对下行压力，经济保持平稳发展。

　　政策扶持积极有效。面对严峻的经济形势，出台支持汽车产业发展、鼓励外贸出口、促进房地产市场健康发展等一系列政策，实施产业龙头促进计划，开展“进企业、摸实情、解难题、促发展”活动，建立领导挂钩重点产业、联系重点企业制度。特别是针对企业融资难、融资贵问题，出台鼓励金融服务实体经济、加强中小微工业企业融资服务等政策，设立企业还贷应急资金，创新政银企担风险共担机制，新引进厦门国际银行、泉州银行，中小微企业贷款余额增长12.8%、达到559亿元。全市新增规模以上工业企业137家、亿元企业34家，规模以上工业经济效益综合指数继续居全省首位。

　　重点项目建设加快。坚持抓项目促投资，发挥投资的关键作用。建立项目工作综合考评机制，开展“四个一批”项目推进活动，启动实施2014～2018年重大投资项目推进计划。城乡基础设施“六项提升工程”实施项目267个，完成投资158亿元。220个省、市重点项目完成投资504亿元，占全年目标的136%。60个重特大项目完成投资199亿元，占全年目标的134.7%。新龙马微车、新能源汽车上市销售，新龙马发动机等68个重点项目建成或投产，厦蓉高速龙岩扩容红坊至和溪段全线开工，厦蓉高速龙岩互通迁建春节前可投入使用。组织赴北京、广东、台湾等地开展招商活动，成功对接一批重大项目。以全军政治工作会议在古田召开为契机，深入研究军民融合发展实施意见，成立专门工作班子，积极开展项目对接、企业对接，达成18个意向项目。全年对接“三维”项目147个，总投资673亿元。

　　产业转型升级扎实推进。实施促进产业转型升级和三次产业加快发展等行动计划，调整优化产业结构。工业重点产业持续壮大，机械、有色金属、烟草、能源精化“四大天王”产业实现产值974亿元、增长11.8%，“古田”精品香烟获批生产，烟草产业税收突破100亿元；环保、稀土、新材料等新兴产业加快发展。全市规模以上工业增加值538亿元、增长12.5%，工业投资729亿元、增长13.2%。国家可持续发展实验区通过现场验收，创建国家高新区通过部委审核，被确定为国家知识产权试点城市，龙净环保项目成果获国家科技进步二等奖，全国首家空气污染治理设备产品质检中心获得国家批准，国家级矿产品检测重点实验室通过验收。建筑业实现增加值170亿元，增长10.4%。现代农业加快发展，农林牧渔业增加值192亿元、增长4%，粮食生产连续七年增长，林下经济产值突破100亿元、增长105.8%，花卉苗木产值40亿元、增长25%，农副产品加工业产值242亿元、增长17.8%，漳平木村林产和武平新洲林化被确定为首批国家林业重点龙头企业。现代服务业稳步增长，第三产业增加值551亿元、增长8%，新增限额以上商贸企业676家、累计1546家。全年接待游客2161万人次，旅游总收入166亿元，分别增长19.5%和24.5%，古田景区通过5A级景观质量评审，连城冠豸山获评国家生态旅游示范区，永定获评2014“美丽中国”十佳旅游县。

　　（二）城乡建设力度加大，生态环境不断优化。

　　宜居环境建设顺利实施。523个项目完成投资122亿元，占全年目标的122.4%，居全省第三位。完成43个城镇“三边三节点”整治提升，新建改造给排水、燃气管网370公里。“美丽乡村”建设有序实施，完成10个社区、128个村庄整治，历史文化名城名镇名村、传统村落保护进一步加强。拆除“两违”建筑507万平方米，“三化三不留”制违工作经验在全省推广。

　　城市功能更加完善。积极推进新型城镇化，永定撤县设区获得批准，全市城镇化率52%，比上年提高1.1个百分点。中心城市教育文体、医疗卫生、城市交通等专项规划进一步提升；理顺市区征迁工作机制，十大片区改造稳步推进，解放北路延伸段、北市场停车场、登高山和莲花山连接栈道桥等项目建成；新投入公交车50辆，新增绿地156公顷；建成区面积扩大3平方公里、达到48平方公里。县域城市建设力度加大，“321”特色乡镇建设扎实推进，高陂镇入选全省首批“小城市”培育试点。

　　生态建设成效明显。完成水土流失治理55.3万亩，占任务的117.4%，提前超额完成“十二五”治理任务。造林绿化15.3万亩，占任务的123.2%。连续15年实现耕地占补平衡。全市新关闭生猪养殖场2699户、拆除猪舍68万平方米，削减生猪存栏38万头；黄岗水库上游生猪养殖场关闭拆除全面完成。实行“河长制”，理顺龙津河管理职责，落实流域治理责任，重点流域水质进一步改善。关停落后产能企业10家，淘汰落后产能62万吨。被评为全国首批创建生态文明典范城市，长汀入选全国生态文明示范工程试点县，上杭、连城通过省级生态县命名，永定通过省级生态县考评验收。

　　（三）突出重点领域攻坚，改革创新稳步推进。

　　市、县两级政府机构改革全面完成，事业单位分类改革稳步推进。行政审批制度改革持续深化，取消、下放行政审批和公共服务事项74项，全面取消非行政许可审批事项。行政服务网络实现县乡村全覆盖，市行政服务中心通过国家级综合标准化单位验收。工商登记制度改革基本完成，全市新登记各类市场主体2.7万户，增长24%。开展政府购买社会服务试点，同步公开全市“三公”经费预决算。县级公立医院综合改革全面启动，长汀基层医改“三权下放”机制被省里总结推广。农村综合改革继续深化，构建“五位一体”的林业金融服务平台。被列入全国首批14个、全省唯一的新型职业农民培育示范市。创新山海协作模式，与厦门共建山海协作经济区，完成583亩启动区的征迁及场地平整工作。龙岩陆地港口岸与厦门口岸实现互联互通。参照执行西部地区政策获得中央补助110亿元，增长110%。

　　（四）民生保障更加有力，群众生活持续改善。

　　人民生活得到新提升。全市各级财政的民生支出达153亿元，占全市财政总支出的74.6%，比上年提高18.4个百分点。20件为民办实事项目全面完成。新增城镇就业2.5万人、农村劳动力转移就业4.9万人。造福工程危房改造完成8300户3.6万人，新开工保障性安居工程1.3万套、基本建成1.2万套，解决48万人农村饮水安全问题。城乡居民基础养老金待遇提高至100元、居全省设区市第二位，“三农”综合保险、城乡低保、新农合、城乡医疗救助、农村五保等保障水平进一步提高，全面实行老年人高龄津贴。精准扶贫“九到户”帮扶机制获国家、省充分肯定，长连武扶贫开发试验区完成总体规划。

　　社会事业取得新进展。新罗、永定、上杭通过全国“义务教育发展基本均衡县”评估，龙岩华侨职专被确定为“国家中等职业教育改革发展示范学校”。在全国创新实施高清网络门诊工程，龙岩人民医院病房综合楼建成使用，新引进3家民营医院。稳妥实施“单独二孩”生育政策，实现“国优”先进单位全覆盖。汉剧进校园、周周有戏等文化惠民活动深入开展，新设2家民办博物馆，新增5个国家历史文化名镇（村），“龙岩采茶灯”列入第四批国家级非遗项目。全民健身活动广泛开展，我市运动员在第17届亚运会和第15届省运会上取得好成绩。民族宗教、外事侨务、库区移民、水文、气象、地震、地灾防治、人防、统计、档案、地方志、科普、邮政管理、无线电管理、老年人、妇女儿童、青少年、残疾人以及慈善事业等工作得到加强。

　　社会治理取得新成绩。成功创建第四届省级文明城市，“六五”普法深入开展。“平安龙岩”建设进一步深化，推进社区网格化建设，创新联访联调机制，有效化解信访积案，重拳整治涉爆、涉麻等各类违法犯罪活动。上杭“1345”调解工作机制获中央领导肯定。安全生产形势持续稳定好转，“四项指数”全面下降。国防动员和后备力量建设进一步加强，“双拥”共建持续深化，军政军民团结更加巩固。

　　（五）自身建设不断加强，工作作风进一步好转。

　　扎实开展党的群众路线教育实践活动，深入查摆、严肃整改“四风”突出问题，严格落实中央八项规定精神，制订实施为民务实清廉工作要求“十二条”，完成超标办公用房清理工作，会议、文件分别减少35.9%、22%，“三公”经费下降27%。自觉接受市人大及其常委会的法律监督、工作监督和市政协的民主监督，广泛接受社会监督。办理人大代表建议304件、政协提案375件，全部按期办结。政风行风、廉政建设、行政监察和审计监督不断加强。

　　各位代表，成绩来之不易，这是省委、省政府和市委正确领导的结果，是市人大、市政协和社会各界监督支持的结果，是全市人民团结拼搏的结果。在此，我代表市政府向全市人民，向人大代表、政协委员、各民主党派、工商联、各人民团体、无党派人士、离退休老同志和社会各界人士，向中央和省直驻岩机构、驻岩部队、武警官兵，向关心支持龙岩发展的港澳台同胞、海外侨胞和国际友人，表示崇高的敬意和诚挚的感谢！

　　在看到成绩的同时，我们也清醒地认识到存在的困难和问题，主要是：经济总量还比较小，三次产业结构不合理，产业竞争力和自主创新能力不足；大项目特别是重大生产性项目缺乏，新的经济增长点和财源税源点不多；城乡基础设施配套不完善，部分地区“脏乱差”和城区交通拥堵问题仍然突出；教育、卫生等公共服务水平与群众期望还有一定差距，食品药品安全、安全生产、社会治安等领域还存在不少隐患，扶贫开发任务艰巨；部分工作人员的担当意识、服务意识不强，仍然存在不作为、乱作为和消极腐败等现象。我们将高度重视这些问题，采取有力措施，认真加以解决。

　　二、2015年工作安排

　　各位代表，今年是全面深化改革的关键之年，是全面推进依法治市的开局之年，也是推动龙岩科学发展跨越发展的重要一年。既要清醒认识“三期”叠加、经济下行压力仍然较大的复杂局面，更要看到中央支持福建加快发展、支持原中央苏区振兴发展的重大机遇，尤其是“新古田会议”胜利召开，为我市发展注入新的动力和活力。要适应新常态，把握新机遇，坚定信心，奋力攻坚，确保“十二五”圆满收官，科学编制“十三五”规划，努力实现更有质量更有效益更可持续的发展。

　　2015年政府工作的总体要求是：深入贯彻党的十八大及十八届三中、四中全会和习近平总书记系列重要讲话、来闽来岩考察重要讲话精神，以邓小平理论、“三个代表”重要思想、科学发展观为指导，坚持稳中求进工作总基调，以提高经济发展质量和效益为中心，深化改革开放，突出创新驱动，加强民生保障，振红土地雄风，谋跨越式发展，加快建设“机制活、产业优、百姓富、生态美”的新龙岩。

　　全市经济社会发展的主要预期目标是：地区生产总值增长10.5%左右；地方公共财政收入增长8%左右；全社会固定资产投资增长23%左右；外贸出口总值增长8%，实际利用外资增长6%，内联引资实际利用市外资金增长8%；城镇登记失业率控制在4.2%以内；社会消费品零售总额增长13%，居民消费价格总水平涨幅控制在3%左右；人口自然增长率控制在9.5‰以内；城镇居民人均可支配收入增长10.5%，农民人均可支配收入增长11%；完成省下达的节能减排任务。

　　重点抓好八个方面的工作：

　　（一）深入实施科学发展跨越发展行动计划。

　　抓好重大投资项目。全力抓好“2014～2018年重大项目推进计划”，加快推进261个省、市重点项目建设。突出抓好30个重大工业项目、165个重点工业新增长点项目，工业固定资产投资800亿元，其中技改投资400亿元。新罗龙泰新能源一期、上杭太阳铜业特种电线电缆、武平正德光电二期、中汽宏远客车、紫荆脱硝催化剂等项目建成或投产，新罗森宝异地技改、武平12万吨不锈钢冷轧生产、长汀清华紫荆永磁电机、百雀羚南方生产基地、漳平特钢铸造基地等项目开工建设。加快基础设施建设，赣龙铁路扩能工程、漳永高速、古武高速武平城关至东留、龙长高速古城互通等建成通车，加快推进南三龙铁路、厦蓉高速龙岩扩容工程建设，扎实推进浦建龙梅铁路、龙岩火车站北站房综合枢纽、漳龙铁路漳平至铁山洋段电化改造、古武高速永定至上杭段、厦蓉高速龙岩东联络线等项目前期工作，争取早日开工建设。

　　加大招商引资力度。坚持“走出去”开展大招商、招大商，深化“三维”项目对接。整合招商资源，改进招商方式，推行点对点产业招商、小分队招商、以商引商，实施岩商“回归工程”，拓宽招商渠道。加强项目跟踪对接，持之以恒、锲而不舍抓落实，促进意向项目早签约、签约项目早开工。充分发挥“新古田会议”后续效应，在引进央企、军民融合项目上取得重大突破。加强项目策划开发和储备，做深做实项目前期工作，提高项目对接率、成功率。

　　完善督查推进机制。建立重大项目“一月一协调、一季一督查”和领导挂钩、部门联动、动态调整、综合考评等机制，把工作项目化、项目节点化、节点责任化。落实项目用地、用林，加强要素保障。创新融资方式和渠道，积极推行政府和社会资本合作模式。

　　（二）加快推进产业转型升级。

　　提升壮大优势产业。实施“四大天王、八大金刚”产业产值倍增计划，着力延伸产业链、衍生新产品、拓展新服务，促进产业优化升级。机械产业重点推进新龙马客车、特种专用车以及福龙马环卫装备等项目，加快新能源汽车、烟气脱硫脱硝、有机废气净化等关键领域的研发步伐，力争实现产值350亿元。有色金属产业重点围绕打造采选冶炼、精深加工和虚拟经济“三个紫金”，加快推进紫金铜业铜冶炼技改、中宇有色金属及水暖卫浴生产、金龙稀土特种金属及合金等项目，支持上杭打造全省最大的低压电线电缆出口基地，力争实现产值320亿元。烟草产业重点推进烟厂一区生产线技改、片烟醇化库建设等项目，做大做响“古田”香烟品牌，提升产品附加值，力争实现产值150亿元。能源精化产业重点发展磷化工、碘化工、氟化工与白炭黑等高附加值精细化工产业，加快推进稀土催化剂等项目建设，力争实现产值350亿元。新兴产业重点推进武平正德光电、连城赛特新材、永定德晖照明等一批新兴产业项目，大力培育新型显示、光电新材料、新能源、节能环保等战略性新兴产业。同时，抓好马坑铁矿采选扩建、新罗喜鹊家纺等100个重点技改项目，推动建材、煤炭、钢铁、纺织等传统产业加快调整升级。加强工业园区建设，培育发展汽车、稀土、新型显示等特色产业园区。

　　强化创新驱动发展。高标准推进国家高新区建设。深入实施高新企业成长助推计划，组建科技成果转化基金，推进工业化信息化融合。抓好清华紫荆创新研究院、福建龙腾新能源汽车研究院、市科技创业园二期等创新平台建设，建成5家以上科技企业孵化器。健全创新激励政策，推动优秀技术人才、科研成果与本市企业对接和优化组合，激发社会创新活力。

　　加大企业帮扶力度。深入实施产业龙头促进计划，壮大龙头企业，支持中小微企业加快成长，新增规模工业企业100家以上。推广“手拉手”活动，促进关联企业协作配套，支持企业兼并重组，提升整体竞争力。加强政银企担对接，支持企业直接融资，创新互联网金融服务，拓宽企业融资渠道。

　　（三）大力发展特色现代农业。

　　做强做优特色农业。加强耕地保护，落实粮食种植面积270.5万亩，稳定粮食产量。加强“一区两园”建设，提升农业生产规模化、集约化、高效化水平。大力发展花卉苗木、茶叶、果蔬等特色产业，林下经济实现产值120亿元。加快发展设施农业，抓好50个新扩建设施农业项目，创建省级以上园艺作物标准园8个、畜禽标准化示范养殖场15个。大力发展肉制品加工，实现农副产品加工产值270亿元。加强农产品市场营销，进一步做大连城白鸭、上杭槐猪、长汀河田鸡等特色产品，打造“闽西八大鲜、八大珍”特色品牌。

　　激活农业经营机制。进一步提升新型职业农民培育水平，扶持发展市级以上示范农民合作社70家、示范家庭农场60家。鼓励耕地向专业大户、家庭农场、农业龙头企业流转，发展适度规模经营。总结推广连城县农村金融改革试点经验，创新农村金融服务方式，扩大农业贷款担保基金，加快建立林业金融服务中心。

　　提高农业生产能力。继续推广农业“五新”技术，农作物耕种收综合机械化水平达到45%以上。加强重大农田水利等基础设施建设和管理，重点实施2条流域防洪工程、7条重点中小河流治理、18座小型水库除险加固、8.7万亩农业综合开发和3个中央小型农田水利重点县项目，新开工4个烟草水源工程。

　　（四）培育发展现代服务业。

　　大力发展生产性服务业。继续培育商贸流通重点企业，引进国内外大型知名商贸企业、物流公司、快递公司。加快推进农产品物流交易城、陆地港综合物流中心、龙门物流园等项目建设，推动农村物流、城市配送、冷链运输等物流发展，闽西汽贸城、闽西建材城竣工开业。适应互联网经济，大力发展电子商务，加快推进电商产业园、淘宝·龙岩馆和永丰电子商务园等项目建设，促进线上线下融合发展。加快金融商务中心建设，支持发展融资性担保机构、小额贷款公司和融资租赁公司，鼓励金融机构扩大信贷投放规模、创新金融产品，加大对实体经济支持力度，提高金融服务水平。大力培育商务服务、服务外包、设计咨询、会展经济等新兴产业，鼓励更多企业总部、交易平台进驻龙岩。

　　积极发展消费性服务业。以“古田会议”品牌为龙头，整合红色、客家、生态资源，促进文化与旅游、科技、金融深度融合，推进文化产业繁荣发展。加快国家智慧旅游试点城市和省级旅游综合改革试点建设，稳步推进长汀国家历史文化名城、永定客家古镇等项目建设和冠豸山创建5A级旅游景区工作，确保古田5A级旅游景区获批。强化房地产市场监管，科学有序供应土地，鼓励货币补偿商品房安置，促进房地产市场健康发展。加快培育发展文化创意、医疗保健、养老健康等新兴服务业。多渠道增加居民收入，积极培育信息服务、教育文体和健康养老等消费新热点，满足群众多元化消费需求。

　　（五）积极推进新型城镇化。

　　优化城镇规划布局。按照“全域龙岩、多规融合”的方向，推进国民经济与社会发展规划、城市总体规划、土地利用规划、生态环境保护规划等“多规合一”。优化城市空间布局，科学确定城镇功能定位，形成以中心城市为龙头、县域城市为骨干、中心镇为依托的新型城镇体系。围绕推动“人进城、建好城、管好城”，提升城市承载能力，完善城市服务功能，加快构建“现代骨、传统魂、自然衣”的新型城乡，全市城镇化率提高1.5个百分点以上。

　　深化宜居环境建设。安排宜居环境建设项目530个、总投资92亿元。促进“点线面”项目逐步连线成片，实施38处传统村落保护利用项目，完成130个村庄环境整治，打造12个美丽乡村示范村、1个宜居环境建设示范县；整治城镇“三边三节点”8个，建成市政管网350公里、绿道100公里；改造各类棚户区5000户以上；九龙江、闽江、汀江流域1公里范围内和土楼保护区内66个乡镇污水实现有效处理。

　　做大做美中心城市。完善中心城市片区规划、重大项目建设规划，优化中心城区空间结构。抓好十大片区征迁改造攻坚，有序推进安置房项目建设。加强“一环一轴四纵五横”主干路网建设，打通兴业路一期、龙岩大道南段一期、华莲西路一期等断头路；加快推进西外环、龙腾北路五期、双永高速龙岩北互通连接线、犀牛路一期、北三环、金鸡路等建设。加快停车场、人行地下通道、排水排污、园林绿化等市政建设，从严治理交通拥堵、卫生死角、渣土车违规以及乱倒垃圾弃土等突出问题。保持制违高压态势，确保违法建设“零增长”。加大永定基础设施、公共服务等投入，确保县改区平稳过渡。

　　创新城镇发展机制。深化户籍制度改革，放开城镇落户限制，落实流动人口居住证制度，最大限度吸纳农业转移人口在城镇落户。引入社会资本参与城市管网等准公益性项目投资和运营。进一步完善征迁工作机制，推进依法征迁、和谐征迁。扎实推进高陂、古田等8个省、市镇级“小城市”培育试点建设，继续推进强镇扩权和乡级行政区划调整。

　　（六）巩固提升生态质量。

　　加强源头保护。以建设全国生态文明建设试点为抓手，强化生态理念，进一步加强生态保护。完成造林绿化18.6万亩、森林抚育20万亩，实施封山育林200万亩，做好林地保护管理和森林防火工作。加强集中式饮用水源保护，有序推进水源保护区及上游群众搬迁安置，做好万安溪调水工程前期工作。力争创建国家森林城市、省级生态市，国家环保模范城市通过省初评，新罗、武平通过省级生态县（区）验收。

　　强化综合治理。实施新《环境保护法》，加大环境执法力度。完成水土流失治理43万亩，治理崩岗100个，综合整治坡耕地1万亩。强化流域污染治理，加强水质动态监测，重抓雁石溪、象洞溪、黄潭河综合整治，促进流域水质持续提升。关闭、拆除禁养区畜禽养殖场，加快推进规模化畜禽养殖场全过程综合治理。加强农村面源污染治理。完成27家6万吨/年以下规模煤矿企业关闭任务，做好矿区生态修复。加强机动车尾气污染防治，加快淘汰黄标车和老旧汽车。推行绿色施工、全封闭施工，防止扬尘污染。

　　节约能源资源。大力发展循环经济、生态型经济，推进绿色低碳可持续发展。全面完成省政府下达“十二五”节能减排任务。深入开展水泥、铁合金行业能效对标工作，加强重点用能企业管理，实施30项节能和循环经济重点项目。推进企业开展资源综合利用和清洁生产，7家企业通过省级循环经济示范企业验收。积极推动龙雁经济开发区争创国家循环化改造示范试点园区。

　　（七）深化改革开放。

　　推进重点改革攻坚。进一步简政放权，加快推进行政服务标准化建设，深化行政审批制度改革，加快构建全流程网上办事统一平台，组建市公共资源交易中心。稳步推进行政类和生产经营类事业单位改革，完成市级行政机关与所办（属）企业和经营性资产脱钩。进一步深化投资体制改革，最大限度减少项目审批、资质认定和行政事业性收费，持续推进工商注册便利化。设立产业基金，吸引社会资本投入。深化国有企业改革，积极发展混合所有制经济，推进龙岩高岭土公司改制上市。完善开发区管理体制。推进预算管理制度改革，实施“零基预算”和全口径预算管理，逐步建立跨年度预算平衡机制，建立政府性债务风险预警、化解机制。深化文化体制改革，组建市文化发展集团。总结推广长汀基层医改经验，深入推进县级公立医院综合改革，启动实施城市公立医院综合改革。深化农村改革和集体林权制度改革，全面完成211个村土地承包经营权确权登记颁证和2个村农村集体产权制度改革试点工作。积极推进不动产统一登记。

　　全面拓展区域合作。深入实施“东张西望”战略，加快发展开放型经济。加强与东南沿海的衔接，推动厦漳泉龙一体化发展，承接沿海产业梯度转移，发展临港经济。编制厦门龙岩山海协作经济区总体规划，加快基础设施建设，吸引一批龙头企业入驻。深化与赣南苏区对接合作，拓展落实中央支持苏区发展政策。加强与台港澳侨交流合作，提升漳平台湾农民创业园建设水平，推进与台湾现代农业、精密机械、光电信息等产业的深度对接。继续抓好对口援疆援藏工作。

　　大力发展对外经贸。积极培育龙头出口企业，支持新龙马微车等本地产品出口，鼓励紫金矿业、龙净环保等企业“走出去”，拓展国际市场。推动外贸综合物流基地建设，完善陆地港保税仓储功能，促进口岸通关便利化，提升陆地港的口岸功能效应。积极跟进对接福建自贸区、“21世纪海上丝绸之路”建设。

　　（八）保障和改善民生。

　　办好惠民实事。实施22件为民办实事项目，使发展成果更多更公平惠及全市人民。新建7所公办幼儿园；推进基础教育信息化建设，基本实现中小学“班班通”；解决10.7万人农村饮水安全问题；新开工建设保障性安居工程1.2万套，基本建成9000套；完成10个城乡集贸市场标准化建设改造；中心城市新增100辆纯电动新能源公交车；实施“希望工程”、“菜篮子”工程等项目。

　　增强社会保障能力。实施更加积极的就业创业政策，新增城镇就业2.2万人。健全工资正常增长和支付保障机制。城乡居民社会养老保险参保率90%以上，基础养老金待遇提高到每人每月110元以上。继续提高城镇居民医疗保险和新农合筹资标准。进一步完善社会救助制度体系，提高城市低保和特困供养人员保障标准，农村低保标准提高到不低于每人每年2300元。创新居家养老服务机制，推行福利中心公建民营和养老机构民办公助，推进老年活动中心和老年大学建设。保障妇女儿童、青少年合法权益，支持发展残疾人事业和慈善事业。

　　加大扶贫开发力度。推进长连武扶贫开发试验区建设。实施造福工程危房改造8000户，新建46个省市集中安置区、120个10户以上安置点。继续实施“九到户”精准扶贫，整村推进扶贫开发，支持31个重点乡镇、380个贫困村培育特色产业，帮助贫困户发展生产，增强“造血”功能，完成减贫2.74万人目标。扶持革命基点村、少数民族村加快发展。

　　提升公共服务水平。推进义务教育均衡发展，推行城区义务教育“小片区管理”，加大农村薄弱学校“委托管理”试点力度。加快中心城区教育资源整合，推进龙岩一中分校、东山第一小学等项目建设，龙岩外国语学校建成招生。推动高校特色内涵式发展，确保闽西职业技术学院国家骨干高职院校通过验收。加强社区卫生服务中心和乡村卫生院建设，鼓励和规范社会资本兴办医疗机构。强化食品药品安全监管，建立食品可追溯体系，治理餐桌污染。坚持计划生育基本国策，促进人口长期均衡发展。有效整合公共文化资源，加强文化精品创作，继续推进国家级闽西客家文化生态保护实验区创建工作。推进全民健身和竞技体育协调发展，承办好全国首届青运会举重比赛。

　　加强社会治理创新。加强全民普法，弘扬宪法精神。全面推进“平安龙岩”建设，深化平安县乡村三级联创活动，提升联访联调工作水平，加强基层基础建设和社会治安综合治理，依法处理信访事项，加大打击黑恶势力等违法犯罪力度，确保社会安定有序。全面落实安全生产“党政同责、一岗双责”，继续推进安全生产标准化建设提升工程“三年行动”，深入开展重点行业、重点领域专项整治，坚决遏制重特大安全事故。加快公共安全预警防控和应急处置体系建设，提升防灾减灾能力。加强社区建设，做好村（居）委会换届选举工作。加强国防动员和民兵预备役建设，支持驻岩部队现代化建设，争创“全国双拥模范城（县）”实现四连冠。做好新形势下外事侨务、民族宗教工作。

　　三、全面加强自身建设

　　大力弘扬古田会议精神、才溪乡调查精神，严格落实为民务实清廉工作要求“十二条”，做到为人民服务、对人民负责、受人民监督，努力打造人民满意的法治政府和服务型政府。

　　全面推进依法行政。把政府工作全面纳入法制轨道，坚持在党的领导下，自觉接受人大及其常委会的法律监督、工作监督和政协的民主监督，依法全面履行政府职能。探索推行“权力清单、责任清单、负面清单”制度，确保“法无授权不可为、法定责任必须为、法无禁止均可为”。建立健全重大决策合法性审查机制、重大决策终身责任追究制度及责任倒查机制，加强行政复议、行政应诉和执法监督。全面推进政务公开，让行政权力在阳光下运行。

　　更加突出务实为民。始终将民生诉求作为政府工作的第一信号，坚持“四下基层”，访民情、听民意、解民忧，实实在在为群众谋福祉。以更高的标准、更严的措施，深入抓好“四风”突出问题的整改，坚决纠正损害群众利益的不正之风。大力压减“三公”经费等一般性支出，优先保障教育、卫生等民生事业发展。

　　着力优化发展环境。强化担当意识、责任意识和效率意识，对分内之事马上就办，对困难之事攻坚克难，对棘手之事善于破解。强化效能督查，严格行政问责，大力整治“庸懒散拖”，切实解决“不作为、慢作为、乱作为”的问题。以政务诚信带动商务诚信、社会诚信，努力创造更加公平开放、规范有序、充满活力的发展环境。

　　始终保持清正廉洁。严格落实党风廉政建设责任制，增强勤政廉政意识，筑牢思想道德防线。真正把党纪政纪当成高压线、警戒线，保持敬畏之心，扎紧制度笼子，用制度管权、管钱、管事。严格执行中央八项规定精神，厉行勤俭节约，反对铺张浪费。强化行政监察和审计监督，坚决从严查处违法违纪案件，用严的制度和铁的纪律打造过硬干部队伍。

　　各位代表，面对新使命、新征程，我们责任在心、重任在肩。让我们紧密团结在以习近平同志为总书记的党中央周围，在省委、省政府和市委的坚强领导下，抢抓机遇，开拓创新，奋勇拼搏，为在更高起点上推进龙岩科学发展跨越发展而努力奋斗！
